# Longitudinal association between negative life events and depression among Chinese senior high school students: the mediating role of psychological inflexibility and the moderating role of meaning in life

**DOI:** 10.3389/fpubh.2026.1898663

**Published:** 2026-09-11

**Authors:** Zhendong Wan, Min Cao, Shuanghu Fang, SiLiang Yang, Xinpei Zhang

**Affiliations:** 1 School of Educational Science, Anhui Normal University, Wuhu, China; 2 School of Education, Hunan First Normal University, Changsha, China

**Keywords:** Chinese senior high school students, depression, meaning in life, negative life events, psychological inflexibility

## Abstract

**Background:**

Negative life events are a risk factor for depression among Chinese senior high school students. Psychological inflexibility may mediate this relationship, whereas meaning in life may serve as a buffer. Prior cross-sectional studies lack temporal evidence; this two-wave 3-month time-lagged design preliminarily tests sequential moderated mediation, whereas multiwave data support stronger causal inference.

**Methods:**

A longitudinal survey with 3-month intervals was conducted among 940 senior high school students in Hunan Province, China. The participants completed measures assessing the Negative Life Events Scale, Psychological Inflexibility Scale, Meaning in Life Scale, and Depression Scale. A moderated mediation model was used to explore the relationships among these variables.

**Results:**

The results showed that (1) negative life events (T1) positively predicted depression (T2) [β = 0.20, *SE* = 0.02, *p* < 0.001, 95% *CI* (0.13, 0.28)]; (2) psychological inflexibility (T2) mediated the relationship between negative life events (T1) and depression (T2) [the indirect effect was 0.03, *SE* = 0.01, 95% *CI* (0.01, 0.05)]; and (3) meaning in life (T1) moderated both the first stage of the mediation pathway [β = −0.10, *SE* = 0.02, *p* < 0.001, 95% *CI* (−0.15, −0.05)] and the direct path [β = −0.11, *SE* = 0.02, *p* < 0.001, 95% *CI* (−0.17, −0.04)]. Specifically, higher levels of meaning in life were associated with weaker positive predictive associations between negative life events and both psychological inflexibility and depression.

**Conclusion:**

These findings highlight psychological inflexibility as a mediator and meaning in life as a protective buffer against depression among Chinese senior high school students, informing prevention and intervention efforts.

## 1 Introduction

Depression, a prevalent psychological disorder, is characterized primarily by a persistent low mood, diminished interest, and lack of energy and is often accompanied by cognitive, physiological, and behavioral changes (1–3). In recent years, the prevalence of depression among Chinese middle school students has become increasingly concerning, with a trend toward both generalization and younger onset. For example, the 2022 National Depression Blue Book reports that 30.28% of individuals with depression in China are under 18 years old, and half of them are students. A study revealed that approximately a quarter of Chinese middle school students are troubled by depressive symptoms (4). Additionally, a nationwide meta-analysis revealed that the pooled prevalence of depression among Chinese children and adolescents is 26.17%, and the detection rate of depressive symptoms among high school students is as high as 28.23% (5). These findings clearly demonstrate that depression is no longer an isolated phenomenon but rather a serious and widespread issue affecting millions of Chinese middle school students and their families. The consequences of depression are extensive and far-reaching. Academically, it can lead to learning difficulties and a decline in academic performance (6); socially, it may result in withdrawal behaviors (3), impaired social functioning (7), and difficulties with attention and concentration (8). Prolonged depression can also hinder personality development, increase the risk of recurrence in adulthood, and contribute to non-suicidal self–injury (9) or even suicidal ideation (10), causing irreparable harm to families and society. Given that senior high school students bear the highest risk, addressing depression specifically within this senior high school subgroup is both urgent and necessary. Conducting indepth research on this issue holds significant theoretical and practical value for developing effective strategies for early identification and intervention, ultimately safeguarding the mental health of this high-risk senior high school population.

### 1.1 Negative life events and depression

According to the Diathesis-Stress Model, depression results from the interaction between an individual's inherent susceptibility and environmental stressors. When an individual encounters an external stressor, their vulnerable traits, such as negative cognitive schemas or difficulties in emotional regulation, are activated, and they may be unable to cope effectively, leading to depressive symptoms (11). Negative life events are common factors that trigger depression in middle school students (12–14) and pose a significant threat to their mental health during the critical period of self-identity development. Negative life events are defined as experiences that harmfully affect individuals, causing stress, distress, or misfortune, and they often require prompt stress management to address significant life changes (15). Studies focusing on middle school students have confirmed a positive correlation between negative life events and depression: the more negative events adolescents experience, the more likely they are to develop depressive symptoms (13, 16–18). For example, a meta-analysis confirmed a moderate positive correlation between negative life events and depression among adolescents (17). Peng et al. (14) also reported a positive predictive effect of negative life events on adolescent depression in a cross-sectional study of primary and secondary school students. However, most existing studies use cross-sectional designs, which can only verify static correlations and are limited in revealing dynamic mechanisms and causal directions. Moreover, the influence of negative life events on depression may involve delayed effects that single-time-point studies cannot capture, suggesting that their relationship is dynamic over time and underscoring the need for longitudinal tracking. Notably, middle school students undergo dramatic developmental changes and face multiple pressures, including academic and interpersonal factors (19). Senior high school students, in particular, are situated in a unique developmental window while facing an exceptionally intense stressor—the national college entrance examination—which may interact with negative life events to foster psychological inflexibility and increase vulnerability to depressive symptoms. To address these issues, this study adopts a two-wave longitudinal design with a 3-month interval to examine the predictive effect of negative life events on depression in this population.

### 1.2 The mediating role of psychological inflexibility

With respect to the internal mechanism linking negative life events and depression among senior high school students, the emergence of a rigid psychological coping pattern called psychological inflexibility is an important factor to consider. Psychological inflexibility is the opposite concept of psychological flexibility and refers to an individual's inability to adapt his or her thoughts and behaviors flexibly to changing situations (20). It involves a lack of dynamic adaptive responses and includes components such as experiential avoidance (avoiding discomfort or negative experiences) and cognitive fusion (treating negative thoughts as if they directly reflect reality) (20, 21). According to the Relational Frame Theory of psychological inflexibility (22), when faced with adverse external circumstances, individuals tend to confine themselves within a narrow reality constructed by language, which prevents them from maintaining flexibility or focusing on external experiences. Research has shown that middle school students who encounter negative events such as parental rejection, harsh parenting, school rumors, or peer abuse are more likely to engage in experiential avoidance or struggle to respond flexibly to current experiences, resulting in high levels of psychological inflexibility (23–28). Essentially, these various negative experiences constitute negative life events, representing different forms of negative interpersonal relationships. Moreover, the self-regulation abilities of adolescents, particularly senior high school students, are not yet fully developed, making them more prone to experiential avoidance, excessive consumption of attentional resources, and a reduced capacity to respond flexibly to environmental changes. This leads to psychological inflexibility when triggered by non-adaptive external experiences. Therefore, this study hypothesizes that negative life events may induce psychological inflexibility in senior high school students. In turn, higher levels of psychological inflexibility may further contribute to the development of depression. Moreover, according to acceptance and commitment therapy, individuals with psychological inflexibility tend to use strategies such as cognitive fusion and experiential avoidance when facing external stressors. This often results in depressive symptoms, including low mood, loss of interest, feelings of worthlessness, and behavioral withdrawal. Studies have shown that when senior high school students overuse or misapply language rules for self-regulation, they tend to focus excessively on pain, deviate from value-guided behavior, and experience confusion or low meaning in life, which can contribute to depression (29). Research on adolescent depression has also revealed that individuals with high levels of psychological inflexibility are more likely to experience depression and other emotional problems (30–32). Therefore, this study hypothesizes that negative life events may indirectly influence depression in senior high school students through psychological inflexibility.

### 1.3 The moderating role of meaning in life

However, not all senior high school students develop depression or exhibit depressive symptoms when facing negative life events. The risk buffering model suggests that protective factors, such as meaning in life, can mitigate the negative impact of risk factors on individuals (33). In addition, cognitive behavioral therapy (CBT) emphasizes that the meaning individuals attribute to life events or facts helps them cope with external stress and maintain mental health (34, 35). Meaning in life refers to an individual's reflection on and questioning of life-related issues, such as responsibilities, the value of existence, and the essence of being (36). It is a subjective experience of meaning that arises during the process of seeking answers to these questions (37). First, meaning in life may moderate the mediating path of psychological inflexibility. According to the two-dimensional model (38), a complete meaning in life consists of two components: meaning possession and meaning pursuit. The first component can provide psychological resources and reduce mental depletion, whereas the latter helps individuals actively confront challenging and negative life events. Through active cognitive reconstruction, negative life events can be integrated into one's life narrative. Studies (39, 40) have shown that, compared with students with a low level of meaning in life, students with a high level of meaning in life are more likely to pursue meaning in life, understand life values and goals, and face negative life events rationally, which may prevent them from becoming psychologically inflexible. Moreover, research has shown that meaning in life can moderate the pathway through which middle school students engage in negative thinking and subsequently experience negative emotions, such as anxiety (35). Therefore, this study hypothesizes that meaning in life may play a moderating role in both the first and second halves of the mediating path of psychological inflexibility.

Additionally, meaning in life may moderate the direct relationship between negative life events and depression. As a form of comprehensive psychological capital, meaning in life encompasses positive psychological factors such as resilience, optimism, hope, and self-efficacy (41). It enhances adaptive capacity, helping adolescents activate recovery mechanisms when facing negative events and reducing the likelihood of experiencing negative emotions (42). Studies have shown that middle school students with a low level of meaning in life are more prone to negative emotions, such as anxiety (43), whereas those with a high level of meaning in life are more likely to experience positive emotions, such as happiness (35). This protective effect may interact with negative life events to counteract or weaken depressive symptoms. Therefore, this study hypothesizes that meaning in life also plays a moderating role in the direct pathway from negative life events to depression. Building on this, meaning in life as a protective buffer may theoretically moderate both segments of the mediating pathway and the direct association between negative life events and depression. To fully test this full set of theoretical possibilities, we propose three separate hypotheses (H3, H4, and H5) to verify the moderating effect on each single pathway, and the empirical results identify which conjectures gain statistical support.

### 1.4 Present investigation

**Figure 1:**
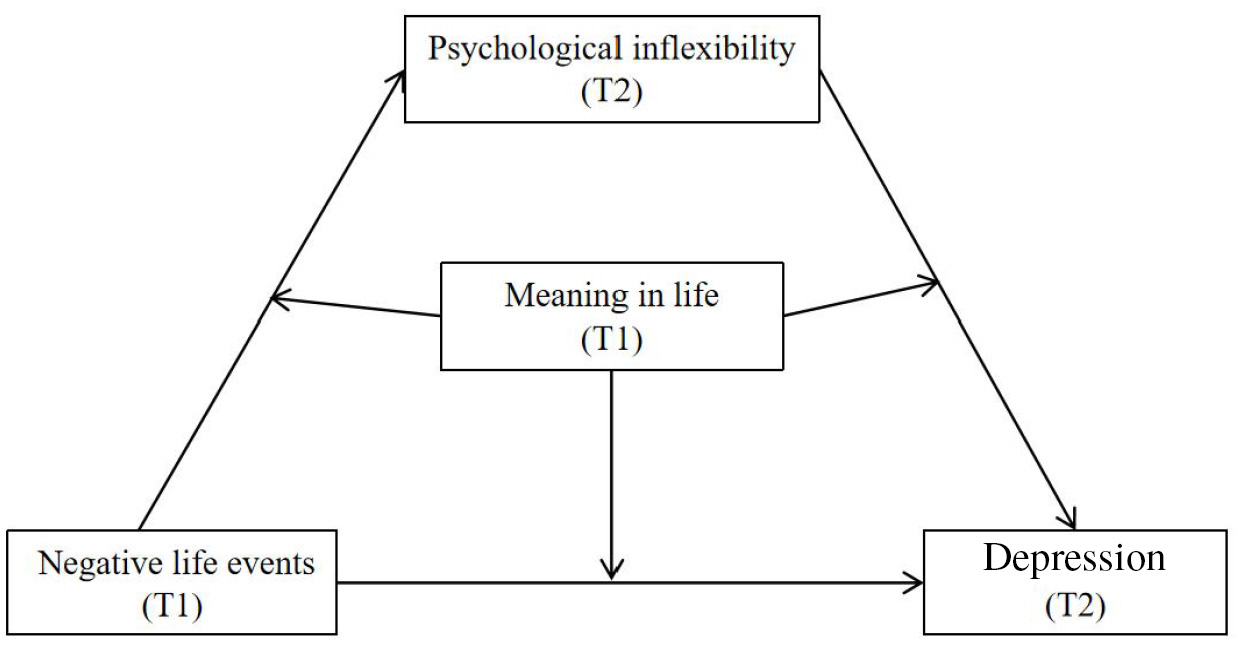
Hypothesized model.

This study constructs a moderated mediation model, as shown in Figure 1, to explore the direct impact of negative life events on depression, examine the mediating effect of psychological inflexibility, and explore the moderating effect of meaning in life. The specific hypotheses are as follows:

*H1* Negative life events (T1) positively predict depression (T2) among senior high school students;
*H2* Psychological inflexibility (T2) plays a mediating role in the longitudinal relationship between negative life events (T1) and depression (T2) among senior high school students;
*H3* Meaning in life (T1) plays a moderating role in the relationship between negative life events (T1) and psychological inflexibility (T2) among senior high school students;
*H4* Meaning in life (T1) plays a moderating role in the relationship between psychological inflexibility (T2) and depression (T2) among senior high school students;
*H5* Meaning in life (T1) plays a moderating role in the relationship between negative life events (T1) and depression (T2) among senior high school students. Notably, these hypotheses cover all potential moderating paths inferred from prior theories without presetting whether each prediction can be statistically validated in the current sample.

## 2 Methods

### 2.1 Participants and procedure

A cluster sampling method was adopted to select second-year senior students (equivalent to 11th grade) from a senior high school in Hunan Province as the subjects. In the Chinese education system, senior high school covers grades 10–12 (typically ages 15–18), and the participants in this study were sophomores (grade 11). The subjects were tracked and tested in two stages with an interval of 3 months, with each class as the unit. The first test was conducted in October 2025 (T1), and 1,086 valid questionnaires were recovered after those with regular responses, missing data, etc., were excluded. The effective rate was 91.2%. The second test was conducted in January 2026 (T2), and 987 valid questionnaires were recovered after those with regular responses, missing data, etc., were excluded. The effective rate was 84.6%. The data from T1 and T2 were combined and processed. Finally, 940 valid questionnaires were matched. There were 487 boys (51.8%) and 453 girls (48.2%), with an average age of 16.46 ± 0.45 years. We conducted chi-square tests to compare gender differences between attrited participants and retained participants, and independent-samples t-tests to examine group differences in age and all study variables. The results revealed no significant gender difference between the two groups (χ^2^ = 1.29, p = 0.26). Likewise, there were no statistically significant between-group differences in age or any study variable (t = 0.10 ~ 0.77, ps > 0.05). These findings indicate that systematic participant attrition was not present in the current study.

The questionnaire link was distributed in computer classes under the supervision of the subject teachers. The test was conducted according to the unified test instructions, and participants were required to complete the questions in the questionnaire within the specified time. If necessary, they could provide explanations to the participants. All the participants were informed of the confidentiality measures and their right to withdraw at any time.

### 2.2 Measures

#### 2.2.1 Negative life events

The Adolescent Self-rating Life Events Checklist (ASLEC) developed by Xin and Yao (44) was adopted to measure the impact of negative life events on senior high school students. This scale consists of 26 items spanning five dimensions, including learning pressure (heavy study burden), relationship pressure (being misunderstood), punishment (criticism and punishment), loss (being away from family), and adaptation problems (urgent illness of relatives and friends). It adopts a 6-point Likert scoring system: 0 = the event did not occur, ranging from 1 = no impact to 5 = extremely severe impact. Higher total scores indicate stronger adverse impacts of negative life events on senior high school students. The Cronbach's α of the scale was 0.95 at T1.

#### 2.2.2 Depression

The Depression Symptom Scale (PHQ-9) developed by Kroenke et al. (45) and revised by Hu et al. (46) was used to measure the depression level of senior high school students. The scale consists of 9 items, such as “lack enthusiasm or interest when doing things”, and uses a 4-point Likert scale (0 = not at all to 3 = almost every day). The higher the total score is, the greater the depression symptom level of the participants. The Cronbach's α values were 0.93 at T1 and 0.92 at T2 in the current study.

#### 2.2.3 Psychological inflexibility

The Adolescent Avoidance and Integration Questionnaire (AFQ) developed by Greco (47) and revised by Chen et al. (48) was used to measure the psychological inflexibility level of senior high school students. This scale consists of 8 items, such as “I am afraid of my feelings”, and uses a 5-point Likert scale (1 = completely inconsistent to 5 = completely consistent), with a higher total score indicating a greater degree of psychological inflexibility. In this study, the Cronbach's α of the scale was 0.89 at T2.

#### 2.2.4 Meaning in life

The Meaning in Life Scale developed by Steger et al. (38) and revised by Wang (49) was used to measure meaning in life of senior high school students. The scale consists of 10 items, such as “My life has a clear direction”, and uses a 7-point Likert scale (1 = completely inconsistent to 7 = completely consistent), with a higher total score indicating a higher level of meaning in life. In this study, the Cronbach's α of the scale was 0.89 at T1.

### 2.3 Statistical analysis

Data processing and statistical analysis, including common method bias tests, descriptive statistics, and correlation analysis, were conducted *via* SPSS 27.0. All continuous variables were standardized prior to model testing, and all reported β values in this paper are standardized regression coefficients. For mediation and moderated mediation analyses, we used PROCESS Model 4 and Model 59, respectively. Bias-corrected 95% *CI*s with 5,000 bootstrap resamples were applied to examine indirect and interactive effects. Questionnaires with straight-lining responses or substantial missing data were excluded at each wave; only participants with complete matched T1–T2 data entered the final analysis.

## 3 Results

### 3.1 Common method variance test

The bifactor model requires adding a method factor as a global factor in addition to the original design factors. If the CFA model with multiple scales combined shows a substantial improvement in fit indices after incorporating the method factor (CFI and TLI increases exceeding 0.1, RMSEA and SRMR decreases exceeding 0.05) (50), it suggests that there was a serious common method bias.

As shown in Table 1, baseline confirmatory factor analysis models were estimated for both T1 and T2 data, followed by bifactor models with an added method factor. For T1, model fit changes were: Δ*CFI* = 0.03, Δ*TLI* = 0.02, Δ*RMSEA* = −0.01, Δ*SRMR* = −0.01. For T2, the changes were: Δ*CFI* = 0.04, Δ*TLI* = 0.03, Δ*RMSEA* = −0.01, Δ*SRMR* = −0.01. In both waves, the improvements in fit indices did not meet the criteria for severe common method bias. Thus, common method bias was not a serious concern in this study.

**Table 1:** Results of the common method bias effect test.

Times	Model	χ^2^/df	*RMSEA*	*CFI*	*TLI*	*SRMR*
T1	*M1:* baseline model	2.90	0.05	0.91	0.91	0.05
	*M2:* bifactor model (*added method factor*)	2.35	0.04	0.94	0.93	0.04
T2	*M3:* baseline model	2.91	0.05	0.91	0.91	0.05
	*M4:* bifactor model (*added method factor*)	2.19	0.04	0.95	0.94	0.04

### 3.2 Descriptive statistics and correlations

The results revealed that negative life events were significantly correlated with psychological inflexibility, depression, and meaning in life (Table 2). Specifically, negative life events (T1) were positively correlated with depression (T1/2; *r* = 0.47, *p* < 0.001; *r* = 0.58, *p* < 0.001), and psychological inflexibility (T1/2; *r* = 0.45, *p* < 0.001; *r* = 0.56, *p* < 0.001) and negatively correlated with meaning in life (T1; *r* = −0.22, *p* < 0.001). Depression (T1) was positively correlated with depression (T2; *r* = 0.78, *p* < 0.001), and psychological inflexibility (T1/2; *r* = 0.29, *p* < 0.001; *r* = 0.39, *p* < 0.001) and negatively correlated with meaning in life (T1; *r* = −0.20, *p* < 0.001). Depression (T2) was positively correlated with psychological inflexibility (T2; *r* = 0.50, *p* < 0.001) and negatively correlated with meaning in life (T1; *r* = −0.23, *p* < 0.001). Correlation analyses showed non-significant associations between gender, age and all core research variables; thus, gender and age were not included as covariates.

**Table 2:** Descriptive statistics and correlation analysis results.

Variables	1	2	3	4	5	6
NLE (T1)	—					
D (T1)	0.47^***^	—				
D (T2)	0.58^***^	0.78^***^	—			
PI (T1)	0.45^***^	0.29^***^	0.39^***^	—		
PI (T2)	0.56^***^	0.39^***^	0.50^***^	0.73^***^	—	
MIL (T1)	−0.22^***^	−0.20^***^	−0.23^***^	−0.13^***^	−0.21^***^	—
gender	−0.02	0.02	−0.01	−0.01	−0.02	0.03
age	−0.01	0.01	0.01	−0.04	0.00	0.05
*M*	1.22	0.66	0.67	2.03	1.93	4.16
*SD*	0.83	0.65	0.65	0.78	0.77	1.45

*N* = 940; ^***^p < 0.001; NLE, negative life events; D, depression; PI, psychological inflexibility; MIL, meaning in life; M, mean; SD, standard deviation.

### 3.3 The moderated mediation model test

First, the variables were standardized *via* the SPSS plugin PROCESS 4.2 model 4. After controlling for psychological inflexibility (T1) and depression (T1), the mediating role of psychological inflexibility (T2) between negative life events (T1) and depression (T2) was tested. The results showed that negative life events (T1) significantly and positively predicted psychological inflexibility (T2) [β = 0.25, *SE* = 0.03, *p* < 0.001, 95% *CI* (0.17, 0.32)]. After including psychological inflexibility (T2) as a mediating variable, the direct effect of negative life events (T1) on depression (T2) remained significant, but the effect size decreased [β = 0.20, *SE* = 0.02, *p* < 0.001, 95% *CI* (0.13, 0.28)]. Psychological inflexibility (T2) significantly positively predicted depression (T2) [β = 0.13, *SE* = 0.03, *p* < 0.001, 95% *CI* (0.06, 0.20)]. This finding indicates that psychological inflexibility (T2) plays a mediating role between negative life events (T1) and depression (T2). The indirect effect was 0.03 [*SE* = 0.01, 95% *CI* (0.01, 0.05)], and this indirect effect accounted for 13.04% of the total effect.

**Figure 2:**
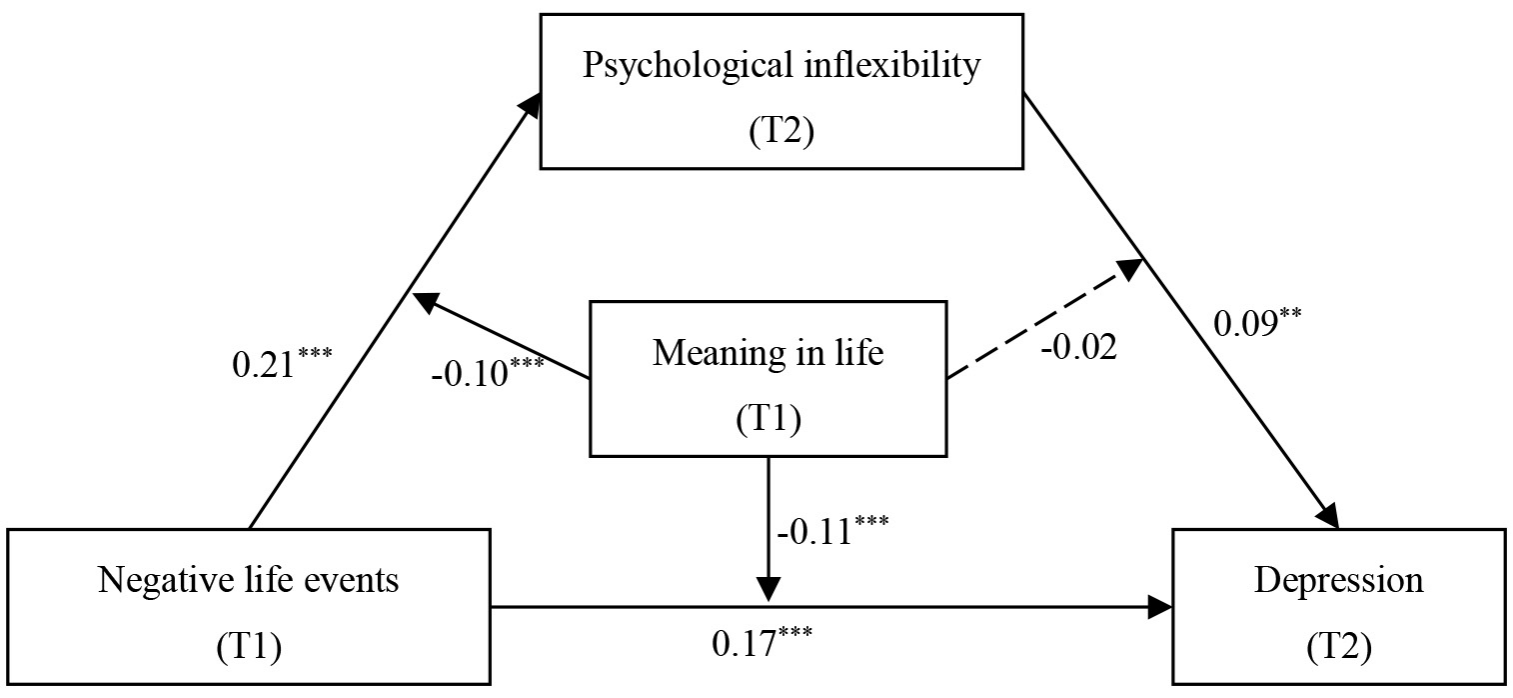
The moderated mediation model of negative life events on depression among senior high school students.

Second, using the SPSS plugin PROCESS 4.2 model 59, the moderating effect of meaning in life (T1) was tested after controlling for psychological inflexibility (T1) and depression (T1). As shown in Table 3, the interaction term between negative life events (T1) and meaning in life (T1) significantly predicted psychological inflexibility (T2) [β = −0.10, *SE* = 0.02, *p* < 0.001, 95% *CI* (−0.15, −0.05)] and depression (T2) [β = −0.11, *SE* = 0.02, *p* < 0.001, 95% *CI* (−0.17, −0.04)]; however, the interaction term between psychological inflexibility (T2) and meaning in life (T1) did not predict depression (T2) [β = −0.02, *SE* = 0.02, *p* = 0.35, 95% *CI* (−0.09, 0.05)]. These results indicate that meaning in life (T1) moderated the relationship between negative life events (T1) and psychological inflexibility (T2), as well as the relationship between negative life events (T1) and depression (T2) (Figure 2). However, it does not play a moderating role in the relationship between psychological inflexibility (T2) and depression (T2).

**Table 3:** Moderating effects of meaning in life.

Variables	PI(T2)			D(T2)		
	β	*SE*	95% *CI*	β	*SE*	95% *CI*
PI (T1)	0.58^***^	0.02	[0.50,0.66]	0.02	0.03	[−0.03,0.08]
D (T1)	0.10^***^	0.02	[0.04, 0.15]	0.62^***^	0.02	[0.55, 0.70]
NLE (T1)	0.21^***^	0.03	[0.14, 0.28]	0.17^***^	0.02	[0.10, 0.24]
MIL (T1)	−0.07^***^	0.02	[−0.11, −0.03]	−0.06^**^	0.02	[−0.11, −0.01]
NLE (T1) × MIL (T1)	−0.10^***^	0.02	[−0.15, −0.05]	−0.11^***^	0.02	[−0.17, −0.04]
PI (T2)				0.09^**^	0.03	[0.03, 0.16]
PI (T2) × MIL (T1)				−0.02	0.02	[−0.09, 0.05]
*R* ^2^	0.62			0.70		
*F*	310.43^***^			306.92^***^		

*N* = 940; ^**^p < 0.01, ^***^p < 0.001; NLE, negative life events; D, depression; PI, psychological inflexibility; MIL, meaning in life.

**Figure 3:**
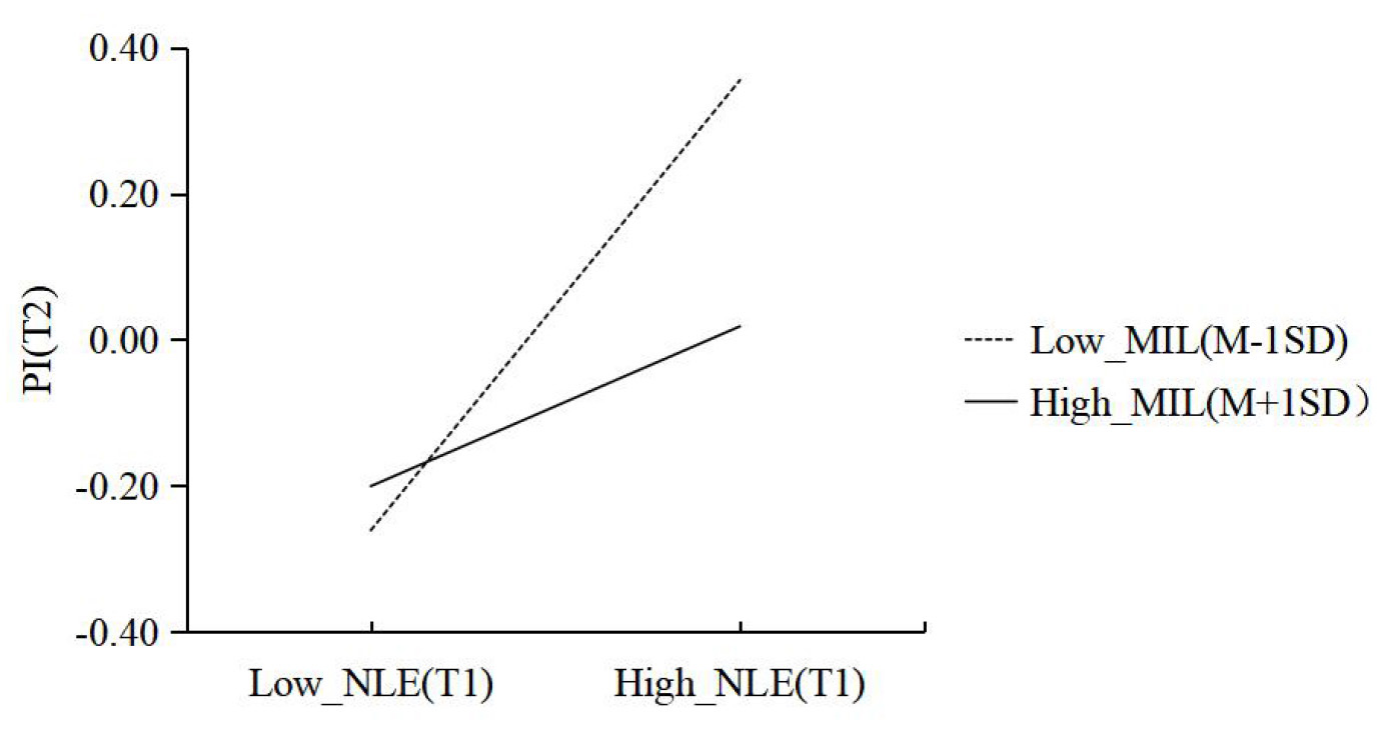
Effect of meaning in life (T1) on the relationship between negative life events (T1) and psychological inflexibility (T2).

**Figure 4:**
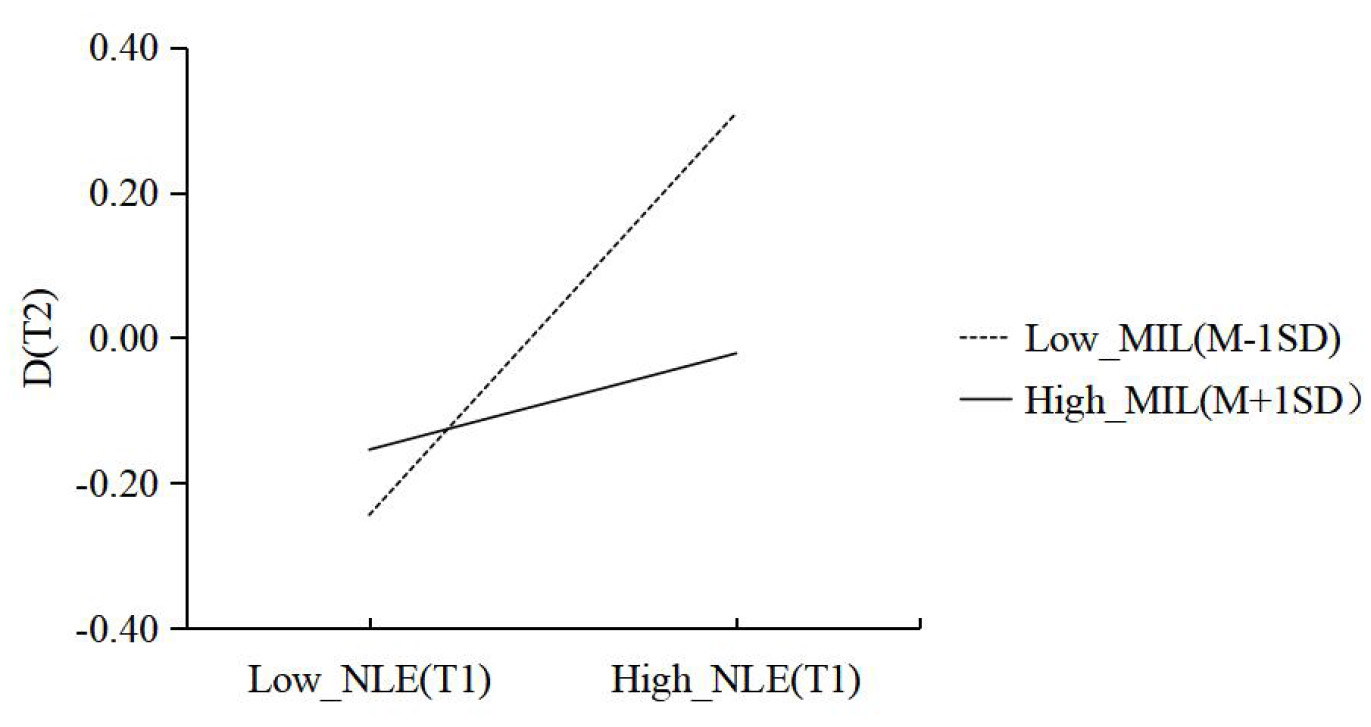
Effect of meaning in life (T1) on the relationship between negative life events (T1) and depression (T2).

The standardized meaning in life was divided into high and low groups according to plus or minus one standard deviation. A simple slope analysis was adopted to explore the essence of the moderating role. A simple slope graph showing the ability of negative life events to predict psychological inflexibility on the basis of different levels of meaning in life perception is shown in Figure 3. The results showed that when meaning in life (T1) was low, negative life events (T1) had a significant predictive effect on psychological inflexibility (T2) [β = 0.31, *SE* = 0.03, *p* < 0.001, 95% *CI* (0.25, 0.36)]; when meaning in life (T1) was high, negative life events (T1) had a significant predictive effect on psychological inflexibility (T2) [β = 0.11, *SE* = 0.04, *p* < 0.01, 95% *CI* (0.04, 0.18)], but the effect was weakened. This finding indicates that the influence of negative life events (T1) has a diminishing effect on psychological inflexibility (T2) and weakens as the level of meaning in life (T1) increases. A simple slope graph showing the prediction effect of negative life events on depression based on different levels of meaning in life perception is shown in Figure 4. The results revealed that when meaning in life (T1) was low, negative life events (T1) had a significant predictive effect on depression (T2) [β = 0.28, *SE* = 0.03, *p* < 0.001, 95% *CI* (0.22, 0.34)]; when meaning in life (T1) was high, negative life events (T1) had a significant predictive effect on depression (T2) [β = 0.07, *SE* = 0.03, *p* < 0.05, 95% *CI* (0.00, 0.13)], but the effect was weakened. This finding indicates that the influence of negative life events (T1) on psychological inflexibility (T2) and depression (T2) both weakened as the level of meaning in life (T1) increased.

## 4 Discussion

This study employed a longitudinal design to construct a moderated mediation model, aiming to explore the impact of negative life events on depression and the underlying mechanisms. The results were as follows: (1) Negative life events (T1) were positively associated with depression (T2); (2) negative life events (T1) indirectly predicted depression (T2) *via* psychological inflexibility (T2); (3) meaning in life (T1) moderated the associations of negative life events (T1) with both psychological inflexibility (T2) and depression (T2); and (4) the association between psychological inflexibility (T2) and depression (T2) was not moderated by meaning in life (T1).

### 4.1 Negative life events and depression

This study confirmed that negative life events were significantly and positively associated with depression among senior high school students, supporting Hypothesis 1. This finding aligns with those of previous studies (14, 16, 51), indicating that the more negative life events students experience, the greater their likelihood of developing depressive symptoms. Furthermore, this result provides additional support for the vulnerability–stress model of depression (11), which posits that psychological disorders are likely to be “triggered” when individuals with high vulnerability encounter sufficiently intense stressors, which may subsequently be associated with depressive symptoms. According to this theory, when encountering many negative life events (such as exam pressure, bullying, parental psychological control, etc.) (12, 52), middle school students with vulnerability traits are prone to activate susceptible factors such as negative cognitive schemas and excessive sensitivity, thereby entering a high vulnerability state of “it is all my fault” and experiencing depressive symptoms (13).

### 4.2 The mediating role of psychological inflexibility

This study also confirmed that psychological inflexibility plays a mediating role in the longitudinal relationship between negative life events and depression among senior high school students, verifying Hypothesis 2. Specifically, the more negative life events (such as academic setbacks or interpersonal conflicts) senior high school students experience, the more likely they are to develop psychological inflexibility, which in turn increases the likelihood of depressive symptoms. This finding further validates both Relational Frame Theory and Acceptance and Commitment Therapy. According to Relational Frame Theory (22), under the influence of an adverse relational framework, individuals tend to avoid negative experiences or develop rigid self-concepts, reducing their flexibility in responding to external events. Therefore, when senior high school students frequently encounter negative life events, such as cyberbullying or parental rejection, they may form maladaptive relational frameworks on the basis of certain contexts and past experiences (e.g., “I am not welcome” or “I am not recognized by my parents”) (23, 53). These frameworks decrease their capacity to respond flexibly to external relationships, leading to psychological inflexibility. Psychological inflexibility, in turn, is associated with depression in senior high school students (54). Students with high psychological inflexibility not only ruminate on self-deprecating thoughts, such as “I am not good enough” or “I am incapable,” but also strongly avoid painful emotions arising from stressful situations. This detachment from their current values prevents them from acting in accordance with their true desires, weakening their coping ability and fostering a sense of helplessness. Consequently, their psychological resources are severely depleted, making it difficult to experience positive emotions, which may ultimately contribute to depressive symptoms, including reduced interest, emptiness, meaninglessness, and loss of vitality. Nevertheless, because psychological inflexibility and depression were both measured at T2, we cannot infer a unidirectional causal flow from psychological inflexibility to depression; the reverse direction or bidirectional influences remain possible. This interpretation should therefore be viewed as a theoretical account requiring further empirical testing with multi-wave designs.

### 4.3 The moderating role of meaning in life

The results of this study indicate that meaning in life plays a moderating role in the first stage of the mediating pathway as well as the direct pathway, supporting Hypothesis 3 and Hypothesis 5. On the one hand, meaning in life moderates the relationship between negative life events and psychological inflexibility (H3). Specifically, when encountering negative life events, senior high school students with high meaning in life are less likely to develop psychological inflexibility than those with low meaning in life. This suggests that meaning in life acts as a protective factor, reducing the likelihood of high psychological inflexibility induced by negative life events. According to the two-dimensional model of meaning in life (38), “having meaning” can provide immediate psychological support, thereby reducing cognitive dissonance and emotional depletion. Moreover, “seeking meaning” motivates individuals to actively adapt to external negative events, promoting cognitive flexibility and actions that are consistent with personal values. Therefore, when senior high school students face negative life events, a high level of meaning in life provides a stable framework, such as viewing setbacks as a normal part of life that helps them respond rationally to challenges and cope effectively, avoiding cognitive breakdown (40). At the same time, the high pursuit of meaning “is like adding meaning-related information schemas” (55), stimulating senior high school students to actively construct meaning, not only finding enlightenment about the value of life in understanding and integrating negative events (such as what I have learned from this) but also adjusting life goals or discovering new values and engaging in behaviors that seek meaning (from focusing on results to focusing on the process).

On the other hand, meaning in life moderates the relationship between negative life events and depression among senior high school students (H5). Specifically, when facing negative life events, students with a high level of meaning in life are less likely to exhibit depressive symptoms. This may be because meaning in life is closely linked to an individual's positive psychological capital (56) and can activate qualities such as resilience, optimism, and self-efficacy, helping students cope with external stressors. From the perspective of meaning in life as a positive psychological resource, it provides values and standards for judgment and offers a sense of control and self-worth in response to life events (57, 58). For senior high school students, a high level of meaning in life can help them reinterpret negative events, for example, viewing an exam failure as a reflection of learning gaps rather than a personal flaw. It can enhance perceived control over related events, such as by adjusting study methods, alleviating feelings of helplessness, and maintaining a stable sense of self-worth. This helps students resist self-denial and avoid depressive symptoms. In summary, the moderating effect of meaning in life on both the first stage of the mediating pathway and the direct pathway further confirms its positive role as an adaptive resource in preventing depression among senior high school students (59, 60).

Interestingly, meaning in life does not moderate the latter half of the pathway from psychological inflexibility to depression; thus, Hypothesis 4 was not supported. In other words, it cannot buffer or reduce the likelihood of depression in senior high school students who are already psychologically inflexible. The explanation may lie in a temporal-stage distinction: meaning in life operates as an early protective resource when stressors initially occur, whereas psychological inflexibility represents a constricted state formed after stress exposure—and once this state is established, meaning in life may no longer be sufficient to counteract it. Consistent with this temporal account, their meaning in life is often state-like rather than enduring, remaining stable only for a short period (61). This transient nature ultimately limits the moderating effect of meaning in life on the relationship between psychological inflexibility and depression. On the other hand, meaning in life is primarily a preventive psychological resource, determining how negative life events are interpreted (62) and providing protection. It operates at the initial stage of stressor impact on an individual's psychology (63). Therefore, possible theoretical interpretation is that meaning in life (T1) may function as an early-stage protective resource, akin to serving as a heuristic “front-end filter,” moderating the early connection between stressors and psychological outcomes in either the first stage of the mediating pathway or the direct pathway. It helps senior high school students reevaluate negative events as understandable, buffering the initial impact of stressors on psychological inflexibility and depression. This timing-based logic is precisely why significant moderating effects emerged for the first-half and direct pathways but failed to emerge for the second-half pathway. However, this “front-end filter” account is a theoretical inference rather than a direct empirical finding from the present data. Given that meaning in life was measured only at T1 and psychological inflexibility/depression at T2, alternative explanations (e.g., that pre-existing depressive symptoms influence meaning perception) cannot be ruled out. Future research using three-wave longitudinal designs is needed to test this temporal sequence more rigorously. In contrast, psychological inflexibility (T2) is a state-like cognitive–behavioral constraint formed after exposure to stress. Once this constricted state develops, depression (T2) may be more likely to co-occur with the inflexible cognitive processing mode. At this stage, meaning in life (T1) might be insufficient, according to this theoretical account, to overcome the existing cognitive confinement, but this possibility should be interpreted cautiously, as our design does not permit causal conclusions about this temporal dynamic.

## 5 Implications and limitations

On the basis of previous studies and the above theories, this study reveals the internal mechanism through which negative life events affect senior high school students' depression through a longitudinal moderated mediation model. It has certain theoretical and practical significance. Theoretically, this study, through a longitudinal model of antecedents (negative life events) and consequences (depression), helps to further understand how negative life events directly and indirectly affect the depression of senior high school students and compensates for the shortcomings of cross-sectional designs. Moreover, this study verifies the mediating role of psychological inflexibility and reveals the mechanism and temporal conditions of the role of meaning in life as a psychological protective factor, providing empirical evidence for acceptance commitment therapy, a two-dimensional model of meaning in life, a risk buffering model, and depression-related theories. In practice, first, the school, family, and community collaborate to build a mental health monitoring network for middle school students; identify early negative life events such as interpersonal conflicts, academic pressure, and family problems; and promptly provide psychological counseling and support. Second, the mediating role of psychological inflexibility suggests that the prevention of depression can be carried out by conducting cognitive flexibility training based on cognitive acceptance commitment therapy, such as teaching students “emotional acceptance” and “thought disengagement” operational skills, encouraging senior high school students to take positive actions on the basis of their own values, guiding students to identify the associations between psychological inflexibility and internal problems, and ultimately avoiding the occurrence of psychological inflexibility. Finally, considering the moderating role of meaning in life, mental health educators can cultivate students' meaning in life through mental health courses. These courses aim to help adolescents establish a stable cognitive motivational emotional system of meaning in life, fully leveraging the role of having meaning in promoting positive change and the role of pursuing meaning in mitigating negative change (64).

Moreover, this study has several limitations. First, all data relied on self-report measures, which may be subject to expectancy effects (65), recall biases associated with current depressive states (66), and social desirability bias, hindering accurate estimation of associations among study variables. Future research could combine data from multiple informants (e.g., teachers, parents, and peers) or adopt experimental methods for cross-validation to improve data accuracy. Second, this study discussed negative life events as a single variable, ignoring the relationships between different dimensions of negative life events and senior high school students' depression and the possible differences. Future research should investigate the impacts of different types of negative life events on depression in detail. Third, although a two-wave longitudinal design was used, both psychological inflexibility and depression were measured at T2, so the temporal precedence of the mediator over the outcome cannot be firmly established. Future research should adopt three-wave or more frequent longitudinal tracking methods to analyze the temporal mechanisms and causal sequence between research variables precisely. Fourth, the sample was drawn exclusively from Hunan Province, which limits the external validity and generalizability of the research conclusions to adolescents in other regions with different socioeconomic and cultural contexts. Future studies should conduct more systematic and multilevel sampling nationwide to improve the external validity of the results. Finally, important demographic variables, such as family socioeconomic status, urban/rural school location, and family background, were not assessed. Future research should include these variables as covariates or moderators in the analysis framework to improve the completeness of the model.

## 6 Conclusion

This study examined the relationships among negative life events, psychological inflexibility, meaning in life, and depression. Consistent with our hypotheses, the results revealed a positive association between negative life events and depression in Chinese senior high school students, and psychological inflexibility was a mediating factor in the effect of negative life events on depression. Meaning in life moderates the relationship between negative life events and psychological inflexibility, as well as the relationship between negative life events and depression, and plays a protective role. Specifically, higher levels of meaning in life were associated with less psychological inflexibility and fewer depressive symptoms among senior high school students facing negative life events.

## Data Availability

The raw data supporting the conclusions of this article will be made available by the authors, without undue reservation.
